# Optimizing Essential Oil Blends by Mixture Design Approaches for Enhanced Antimicrobial and Antioxidant Activity: A Review

**DOI:** 10.1111/1750-3841.70794

**Published:** 2025-12-26

**Authors:** Boutheina Ben Akacha, Miroslava Kačániová, Wirginia Kukula‐Koch, Sandra Cabo Verde, Joana Madureira, Wojciech Koch, Rania Ben Saad, Monika Michalak, Stefania Garzoli, Anis Ben Hsouna

**Affiliations:** ^1^ Laboratory of Biotechnology and Plant Improvement Centre of Biotechnology of Sfax Sfax Tunisia; ^2^ Institute of Horticulture, Faculty of Horticulture Slovak University of Agriculture Nitra Slovakia; ^3^ School of Medical & Health Sciences VIZJA University Warszawa Poland; ^4^ Department of Pharmacognosy with Medicinal Plants Garden Medical University of Lublin Lublin Poland; ^5^ Centro de Ciências e Tecnologias Nucleares (C2TN), Instituto Superior Técnico Universidade de Lisboa Bobadela LRS Portugal; ^6^ Departamento de Engenharia e Ciências Nucleares, Instituto Superior Técnico Universidade de Lisboa Bobadela LRS Portugal; ^7^ Department of Food and Nutrition Medical University of Lublin Lublin Poland; ^8^ Department of Pharmaceutical Sciences, Collegium Medicum Jan Kochanowski University Kielce Poland; ^9^ Department of Chemistry and Technologies of Drug Sapienza University Rome Italy; ^10^ Department of Environmental Sciences and Nutrition, Higher Institute of Applied Sciences and Technology of Mahdia University of Monastir Monastir Tunisia

**Keywords:** antimicrobial activity, antioxidant activity, bioactive compounds, essential oils, mixture design, statistical optimization, synergistic effects

## Abstract

The growing interest in natural alternatives to synthetic additives has driven research into essential oils (EOs) for antimicrobial and antioxidant applications. Statistical mixture design approaches provide effective tools to optimize multi‐component EO blends for enhanced bioactivity. This review presents an overview of recent advances in applying mixture design methodologies, such as simplex lattice and simplex centroid designs, to develop EO combinations with improved antimicrobial and antioxidant activity. We summarize key findings on blend synergies, discuss mechanisms underlying enhanced efficacy, and highlight case studies where optimized mixtures outperform the action of individual EOs. Current patents and practical applications of optimized EO blends are reviewed, and future research directions are proposed, including multi‐component formulations, encapsulation approaches, and machine learning‐driven optimization. By synthesizing these insights, this review highlights mixture design as a sustainable and innovative approach to developing eco‐friendly, effective EO‐based antimicrobial and antioxidant formulations.

## 1 Introduction

The global trend towards natural and environmentally friendly solutions has increased in recent years. According to the World Health Organization, nearly 80% of the world's population already relies on natural products for primary healthcare, while the market for natural food additives is growing at a compound annual growth rate of 6.5% and is expected to reach over USD 50 billion by 2030 (Aware et al. 2022). This demand is driven by growing consumer concern about the health risks of synthetic additives‐ including carcinogenicity and endocrine disruption‐ as well as the environmental impact of their production (Encarnação et al. 2019). In this context, essential oils (EOs) from aromatic plants are emerging as promising natural alternatives thanks to their multifunctional biological activities and high consumer acceptance (Ben Hsouna et al. 2022a; Hsouna et al. 2022; Baj et al. 2023a; Akacha et al. 2023a; Akacha et al. 2025).

Essential oils are complex mixtures of terpenes, phenolic monoterpenes, and oxygenated derivatives that exhibit a broad spectrum of bioactive properties (Angane et al. 2024). Among others, antibacterial activity has been extensively studied, with several essential oils exhibiting strong inhibitory activity against foodborne and clinical pathogens, such as *Escherichia coli* (*E. coli*), *Staphylococcus aureus* (*S. aureus*), and *Listeria monocytogenes* (*L. monocytogenes*) (Akacha et al. 2024b). Mixture optimization studies have shown that the combination of EOs through statistical mixture design can reduce minimum inhibitory concentrations (MICs) by 2 to 4 times compared to single oils (Squeo et al. 2021; Baj et al. 2023b; Angane et al. 2024).

In addition to their antimicrobial potential, EOs have a remarkable antioxidant capacity and play a crucial role in combating oxidative stress, lipid peroxidation, and free radical formation‐ processes that contribute to food spoilage, loss of nutritional quality, and chronic diseases (Aelenei et al. 2016; Ben Hsouna et al. 2024). Recent large‐scale screening revealed that of 423 essential oils tested, 73 exhibited strong free radical scavenging activity, particularly those from the Lamiaceae and Myrtaceae families due to their richness in thymol, carvacrol, and eugenol (Bertin et al. 2020). In addition, optimized blends often show synergistic antioxidant effects and outperform the effects of individual oils. For example, a ternary mixture of *Thymus serpyllum, Mentha pulegium*, and *Mentha piperita* achieved an experimental IC_50_ (half maximal inhibitory concentration) of DPPH (2,2‐Diphenyl‐1‐picrylhydrazyl) value of 0.98 µg/mL, which was significantly lower than the values of the individual oils (Ait‐Ouazzou et al. 2012).

The chemical composition of essential oils is not fixed and may vary substantially between samples. Key drivers of this variability include genetic factors (species and chemotype), environmental conditions (geographic origin, climate, and soil composition), agronomic variables (plant age, phenological stage at harvest, and specific plant part used), and post‐harvest handling. In addition, the extraction technique and parameters (such as steam distillation, hydrodistillation, solvent extraction, and supercritical CO_2_) strongly influence yields and relative concentrations of volatile constituents. Analytical factors and potential adulteration or blending can further modify reported compositions. Because biological activity is often linked to major constituents (such as thymol, carvacrol, eugenol, and linalool), these sources of compositional heterogeneity can substantially affect antimicrobial and antioxidant outcomes; therefore, variability must be considered when comparing studies or proposing optimized blends. However, due to the chemical complexity of EOs, empirical approaches are often not sufficient to fully utilize their potential. Mixture design methods provide a powerful statistical framework to systematically evaluate nonlinear interactions between EO components, identify synergistic antibacterial and antioxidant effects, and determine optimal ratios (Bertin et al. 2020). These approaches enable the development of EO‐based formulations that are more effective, stable, and economical while reducing the risk of toxicity or sensory changes.

Accordingly, this review aims to provide an overview of recent advances in applying mixture design methodologies to optimize essential oil blends for enhanced antimicrobial and antioxidant activity. Accordingly, this review aims to provide an overview of recent advances in applying mixture design methodologies to optimize EO blends for enhanced antimicrobial and antioxidant activity. We summarize key findings on blends’ efficacy, highlight case studies where optimized mixtures outperform single oils, and discuss current patents and applications of these optimized EO blends. For clarity, mixture design methodologies are emphasized as the core focus of this review, highlighting their role in systematically optimizing EO blends for enhanced bioactivity. This structured approach enables systematic exploration of component interactions and harnesses synergistic effects to maximize antimicrobial and antioxidant efficacy. By synthesizing these insights, the review underscores mixture design as a sustainable and innovative strategy for creating effective, eco‐friendly EO‐based antimicrobial and antioxidant formulations.

## 2 Materials and Methods

This review systematically examines the application of statistical mixture design methodologies to the formulation and optimization of EO blends for enhanced antimicrobial and antioxidant activity. Specifically, we focus on studies that employ mixture experiments to identify synergistic EO combinations with improved biological efficacy, and we synthesize their strategies, outcomes, and implications.

An extensive literature search was conducted using multiple databases throughout 2025. This work emphasizes the potential of EOs as sustainable and multifunctional alternatives to synthetic preservatives in the food, pharmaceutical, and cosmetics industries.

The methodology for this review article included a literature search in the following databases: PubMed, Web of Science, Google Scholar, and Scopus. To collect the literature data, several keywords were used, such as “essential oil blends”, “antioxidant activity”, “antimicrobial properties”, “blend design”, “optimization”, and “synergistic effects”. All studies published in peer‐reviewed journals that focused on the antimicrobial properties of EOs, applied blend design methods, and tested antimicrobial efficacy were considered. Data extracted from the selected studies included authorship, year of publication, essential oils used, blend development methodology, and microbial strains tested. The analysis identified common themes, trends, and compelling combinations, assessed each study's methodological rigor, reproducibility, and limitations, and summarized the results to highlight successful applications, best practices, and future research directions. This article provides a comprehensive overview of the current state of research and guides the development of more effective natural antimicrobial agents.

## 3 Common Types of Mixture Designs and Their Implementation

Mixture design, a statistical method for optimizing the composition of mixtures, comprises several types, each suitable for different experimental setups and analytical requirements (Squeo et al. 2021). The most common types of mixture designs and the software included are presented in Table 1:

‐ Simplex lattice design, used to mix experiments with three or more components. All possible combinations of these components are considered in specific proportions. The experimental design is like a lattice structure, which makes it possible to investigate the effects of varying the proportions of the individual components (Belay et al. 2017).
‐ Simplex centroid design, focusing on the centers of the mixture space, which allows for the study of multiple mixtures containing a wide range of components by placing points at the centers of the mixtures. It is beneficial when the number of components is limited (Chen et al. 2010).
‐ Extreme vertices design, typically used when mixtures are studied closer to the boundaries of their components. In this approach, only the vertices (extreme vertices) of the mixture space that correspond to the pure components of the mixture are considered (Menchaca‐Méndez et al. 2022).

**TABLE 1:** Overview of common mixture design types and their strengths and limitations.

Type of mixture designs	Strengths	Drawbacks	References
**Simplex lattice design**	Thoroughly explores all possible combinations of components at specified proportions, providing detailed interaction information.	It can become complex and require many experiments as the number of components increases.	(Duangjit et al. 2014; Belay et al. 2017)
	Effective for identifying synergistic and antagonistic interactions among components.	Computationally intensive and time‐consuming for large mixtures.	
	Well‐suited for optimization and detailed analysis of mixture effects.	It may produce redundant data points in some instances.	
**Simplex centroid design**	Simplify the experimental setup by focusing on centroid points, reducing the required experiments.	It may miss critical interactions present at non‐centroid points in the mixture space.	(Chen et al. 2010; Dias et al. 2019; Rodrigues et al. 2024)
	Efficient for mixtures with a limited number of components.	It is less effective for complex mixtures with many components.	
	Provides a good balance between the number of experiments and the quality of information obtained.	It may not capture the full range of possible interactions between components.	
**Extreme vertices design**	It focuses on the extremes of the mixture space, which can highlight the behaviour of components at their maximum and minimum proportions.	It is limited to studying interactions at extreme points, which may not represent typical use scenarios.	(Yildizel et al. 2020; Akinwande et al. 2023; Attah et al. 2023)
	It helps identify boundary behaviour and performance under extreme conditions.	It may overlook interactions that occur at intermediate proportions.	
	Requires fewer experiments compared to designs that explore the entire mixture space.	It may provide less comprehensive information about the mixture's behaviour.	

Mixture experimental designs possess a distinctive mathematical structure that makes them particularly well‐suited to detect and quantify synergistic or antagonistic interactions among components. Two features are central: (1) the independent variables are component proportions constrained to sum to unity $\left(x_{1}+x_{2}+\cdots +x_{k}=1\right)$, and (2) the response surface is modelled using **S**cheffé‐type polynomials, which are specifically formulated for the simplex geometry of mixture space (Snee and Hoerl 2023).

**FIGURE 1:**
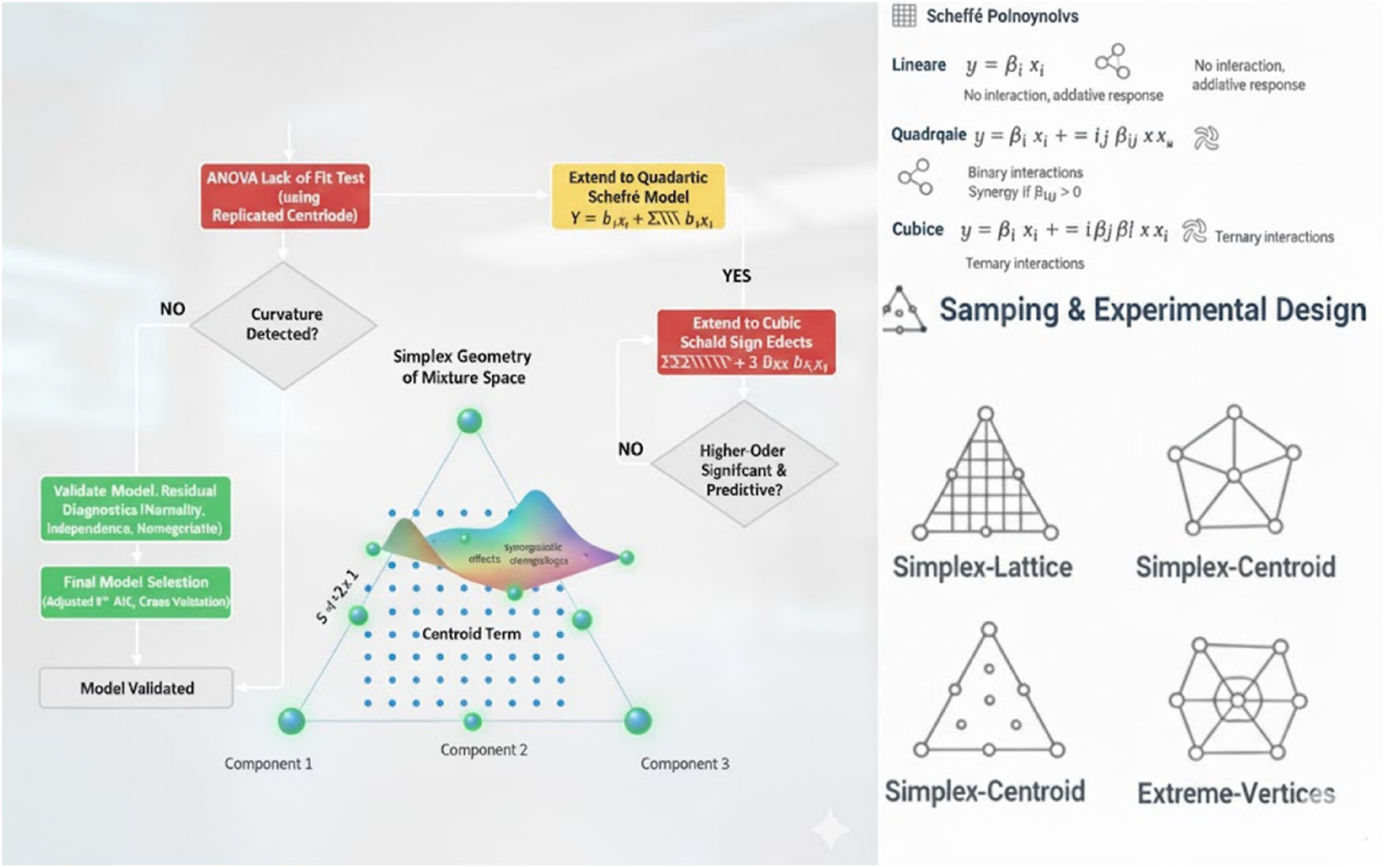
Systematic detection and quantification of synergistic effects via scheffé mixture models.

Scheffé polynomial models are compositionally dependent; standard polynomial models used in factorial designs are inappropriate in Figure 1.

Scheffé polynomials are employed, expressed as follows for a *k*‐component mixture (first degree):

- Linear Scheffé model: *Y = b1x1 ± b2x2 ± b3x3 ± … ± b_qx_q*
  where:
*Y* = response variable
*b_i* = coefficient associated with component *i*
*x_i* = proportion of component i in the mixture.

This model assumes no interaction between components; the response is simply the weighted average of the single‐component effects (Piepel 2007).

- Quadratic (special seconddegree) Scheffé model: *Y = Σ(b_i x_i) + ΣΣ(b_ij x_i x_j), for i < j*
  Expanded form for three components:
*Y = b_1_x_1_ + b_2_x_2_ + b_3_x_3_ + b_12_x_1_x_2_ + b_13_x_1_x_3_ + b_23_x_2_x_3_*
  where:
*b_ij* = interaction coefficient between components *i* and *j* (Piepel 2007).

The pairwise interaction coefficients ($\beta_{ij}$) represent deviations from additivity. A positive $\beta_{ij}$ value (for a response where higher is better) indicates that the mixture of components *i* and *j* performs better than expected from linear additivity, signifying synergism (Okafor and Oguaghabmba 2010).

- Cubic (special thirddegree) Scheffé model: *Y = Σ(b_i x_i) ± ΣΣ(b_ij x_i x_j) ± ΣΣΣ(b_ijk x_i x_j x_k)*, for *i ≤ j ≤ k*
  Expanded form for three components:
*Y = b1x1 ± b2x2 ± b3x3 ± b12x1x2 ± b13x1x3 ± b23x2x3 ± b123x1x2x3*
  where:
*b_ijk* = coefficient for the three‐way interaction between components *i, j*, and *k*

The ternary interaction terms ($\beta_{ijl}$) capture complex three‐component non‐additive behaviors.

Because of the compositional constraint ($\Sigma \;x_{i}=1$), these models do not include an intercept term. The coefficients directly represent the contributions of individual components and their interactions within the simplex (Eleno and Schön 2014). The centroid, defined as the design point where all components are present in equal proportions (x_i​_ = 1/k), represents the geometric centre of the simplex and plays a key role in mixture design analysis. Measuring the response at this point is crucial, as it provides valuable information about the overall curvature of the response surface, allowing nonlinearity to be detected by comparing centroid and vertex responses. Replicated centroid runs also provide an unbiased estimate of pure experimental error, which is required for performing ANOVA lack‐of‐fit testing. Furthermore, centroid observations enable accurate estimation of higher‐order interaction coefficients, particularly in simplex‐centroid designs, where such data enhance the detection of synergistic interactions (Hare et al. 2025; Elbouzidi et al. 2025).

Several design types are commonly used to explore the mixture space and capture interaction patterns. A simple lattice design samples the mixture space on a regular grid of component proportions (e.g., 0, ½, and 1 for a {0, ½, 1} lattice with three components), offering dense coverage of edges and interiors suitable for fitting polynomial models of a chosen order (Piepel 2007). A simplex‐centroid design places experimental points at all vertices, midpoints, and the central centroid, thus focusing sampling on regions most informative for detecting interaction effects while minimizing the number of required experimental runs. When component proportions are subject to practical upper or lower bounds, extreme‐vertices designs are used to sample only the feasible region of the mixture space (Elbouzidi et al. 2025).

Model construction and validation follow an iterative approach. The process begins with fitting the simplest linear model and assessing the lack of fit using ANOVA with replicated points, such as the centroid. If curvature or nonlinearity is detected, the model is extended to include quadratic terms and refitted. Competing models are compared based on adjusted *R^2^*, information criteria, prediction error sum of squares statistics, and cross‐validation performance. Higher‐order terms are retained only when statistically significant (*p < 0.05*) and when they improve predictive accuracy, thereby avoiding overfitting (Chen and Chen 2025a). Finally, residual diagnostics must confirm normality, independence, and homogeneity of variance to ensure model validity.

## 4 Software for Implementing Mixture Designs

**FIGURE 2:**
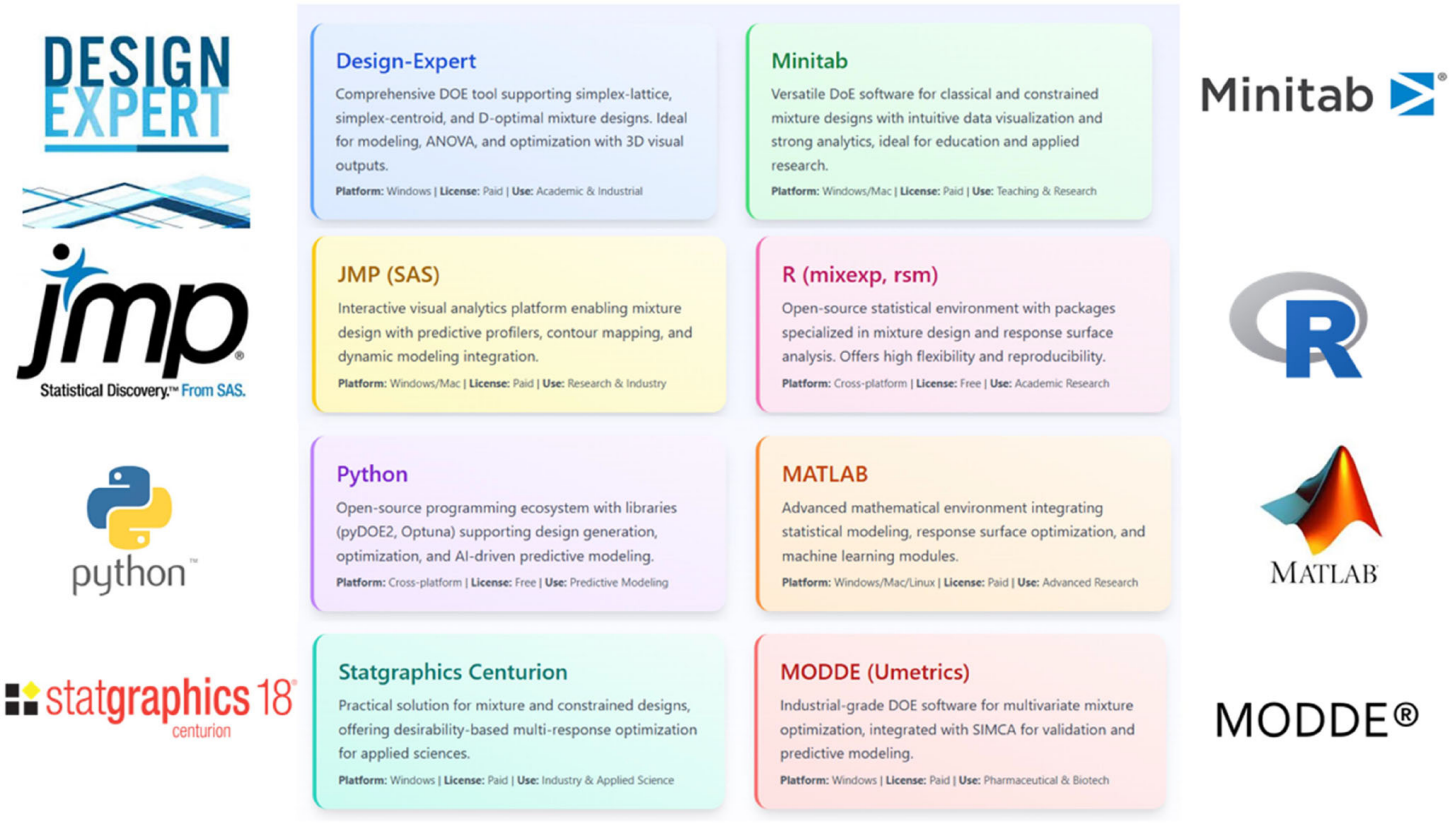
Software tools for implementing mixture designs.

To optimize mixture formulations and analyze interactions between components, several specialized statistical software tools have been developed. These tools facilitate the design, execution, and interpretation of mixture experiments by providing user‐friendly interfaces, robust modelling options, and powerful visualization capabilities (Figure 2).

‐ Design‐expert, a comprehensive solution tailored for designing, executing, and analyzing experiments, including mixing experiments, is particularly suitable for users seeking to optimize mixtures (Ogbonna et al. 2017). It offers diverse experiment design options, such as simplex lattice, simplex centroid, and extreme vertices. It provides intuitive graphical tools to visualize the relationships between mixture components and their effects on responses. Additionally, design‐expert incorporates response surface methodology (RSM) features, enabling researchers to model and optimize their mixtures accurately (Guo et al. 2021). Additionally, it supports the detection of interactions and synergistic effects, making it an ideal tool for in‐depth mixture analysis.
‐ Minitab is powerful statistical software with tools for designing and analyzing mixtures. It is known for its user‐friendly interface and extensive statistical analysis capabilities, making it accessible to beginners and experienced researchers. Minitab offers a variety of options for mixture designs, including simplex grid, simplex centroid, and extreme vertices. Minitab also provides comprehensive data analysis tools that allow users to perform detailed statistical analysis of their experimental results. Minitab's strength lies in its versatility, as it can handle not only mixture experiments but also a wide range of other statistical analyses, making it a valuable tool for researchers working on multidisciplinary projects (Akacha et al. 2024b).
‐ John's Macintosh project (JMP), developed by the statistical analysis system (SAS), is an advanced statistical software that offers robust features for mixture design and analysis (Snee and Hoerl 2016). JMP is mainly known for its dynamic data visualization features that allow users to explore and interact with their data in real‐time. The software supports various mixture design methods, including simplex lattice, simplex centroid, and extreme vertices designs, and offers tools for optimizing mixtures based on multiple responses. JMP's integration with SAS enables more advanced statistical modelling and data analysis, making JMP a preferred choice for complex experimental designs (Paczkowski 2016). In addition, JMP's ability to handle large data sets and its advanced scripting language allow for customization and automation of analysis, providing researchers with greater flexibility and efficiency.
‐ Open‐source environments such as The R Foundation for Statistical Computing (R) and Python have become increasingly popular in academic research due to their flexibility and reproducibility. In R, several packages such as mixexp, rsm, DoE.base, and DoE. MIP array supports the design, modelling, and analysis of mixture experiments. Python, with libraries such as pyDOE2, statsmodels, scikit‐optimize, and Optuna, enables customized design generation, statistical modelling, and integration with machine learning algorithms for predictive optimization. Both platforms are free, cross‐platform, and highly adaptable for research automation (Chen and Chen 2025b).
‐ MATLAB (MathWorks Inc., USA) also provides a powerful environment for modelling and optimization. With its statistics and machine learning toolbox, users can construct customized mixture models, perform nonlinear regression, and visualize response surfaces. MATLAB's integration with machine learning and multivariate analysis modules makes it suitable for advanced data modelling (T. Aditya Sai Srinvias et al. 2023).
‐ Statgraphics Centurion offers a user‐friendly interface for mixture and constrained mixture designs, supporting regression modelling, analysis of variance, and multi‐response optimization using desirability functions. It is a practical choice for academic and industrial applications requiring clear graphical output and statistical rigour (Raissi and Farsani 2009).
‐ MODDE (Sartorius Umetrics, Sweden) integrates seamlessly with SIMCA for advanced multivariate analysis, offering powerful visualisation, model validation, and design space exploration tools. MODDE is widely used in pharmaceutical, biotechnological, and chemical formulation studies where regulatory compliance and process robustness are required.

Other specialized tools, including NemrodW (Université de Montpellier, France) and Statistica (TIBCO Software Inc., USA), also support mixture and response surface designs. NemrodW provides an accessible academic option for basic experimental designs, while Statistica offers a broader analytical environment that encompasses mixture modeling and optimization. Additionally, spreadsheet‐based plug‐ins for Microsoft Excel can be used for preliminary or educational applications, although they are limited in statistical sophistication. The choice of software depends on the scope of the study, the user's statistical proficiency, and resource availability (Guo et al. 2021). Design‐expert and JMP are preferred for comprehensive, publication‐ready analyses; Minitab is ideal for educational and teaching contexts; R and Python offer flexibility and cost‐free reproducibility; and MODDE and MATLAB are best suited for advanced industrial optimization and regulatory environments. The integration of these software tools has significantly improved the precision, reproducibility, and efficiency of mixture design studies applied to the optimization of essential oil blends and other complex natural formulations (Keeling and Pavur 2007; Zieniuk and Bętkowska 2021; Ellouze et al. 2024).

## 5 Synergistic Effects of EOs

Synergy is the phenomenon where the combined effect of two or more components is greater than the sum of their individual effects (Williamson 2001). In the context of EOs, synergy occurs when the blend of different EO components increases the overall antimicrobial, antioxidant, or therapeutic effect compared to each element alone. This concept is particularly precious in optimizing critical oil blends for various applications, such as food preservation, pharmaceuticals, and cosmetics. The idea of synergy is based on the realization that essential oils are complex mixtures of volatile compounds, each with different properties. Combining these compounds can enhance their biological activities through their interactions. Synergistic interactions are critical to maximizing the efficacy of EO formulations as they allow lower concentrations of the individual components while achieving the desired effect, which can reduce potential side effects and costs (Albash et al. 2023).

**FIGURE 3:**
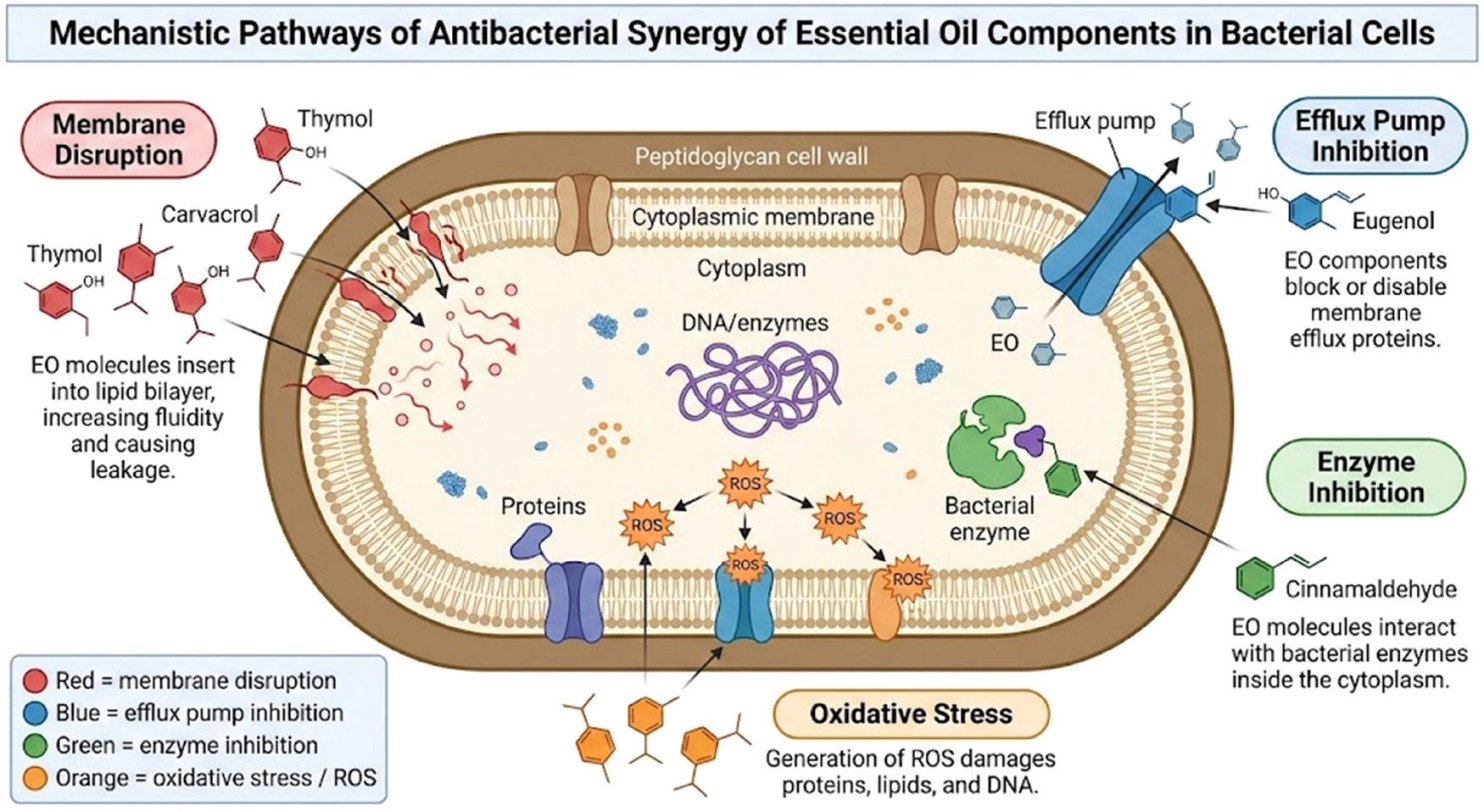
Mechanistic pathways of antibacterial synergy in key essential oil components (eugenol, carvacrol, thymol, and cinnamaldehyde).

The antibacterial activity of EOs results from multiple, interdependent molecular mechanisms that converge to disrupt microbial homeostasis. Phenolic monoterpenes such as eugenol, carvacrol, and thymol, along with cinnamaldehyde, interact with bacterial membranes and intracellular targets, leading to oxidative stress, enzymatic inhibition, and energy depletion. These effects act synergistically rather than independently, amplifying antibacterial potency

(Zengin and Baysal 2014; Ben Hsouna et al. 2017; Akacha et al. 2022b). Figure 3 summarizes these key mechanistic pathways and illustrates their interconnections at both the molecular and cellular levels.

The mechanisms underlying synergistic interactions in essential oils on antimicrobial activity are diverse and can be divided into several categories:

- Pharmacokinetic synergy occurs when one compound increases the bioavailability or absorption of another (Bhattacharya et al. 2021). For instance, one EO component can increase cell membrane permeability, making it easier for another element to penetrate the cell and exert its effect. For example, oils such as oregano and thyme can help improve the effectiveness of certain antibiotics, making them more therapeutic at lower doses (Melo et al. 2015).
- Pharmacodynamic synergy: In this mechanism, the components act on the same or different targets within the microbial cell, resulting in an enhanced antimicrobial effect (Li et al. 2022). For instance, one ingredient may destroy the cell wall while another interferes with intracellular functions, producing a more potent antimicrobial effect.
- Inhibition of enzymes: Some components of EOs can inhibit enzymes responsible for the degradation or detoxification of other elements, thereby prolonging their activity and enhancing the overall effect (Strelow et al. 2004). Eugenol, found in clove oil, can inhibit enzymes like chitin synthase and *β*‐(1,3)‐glucan synthase, which are necessary for the integrity of fungal cell walls. Lavender EO has also been noted for its ability to inhibit various bacterial enzymes (Argüelles et al. 2024).
- Inhibition of efflux pumps: Certain EO compounds can inhibit bacterial efflux pumps, which are responsible for the ejection of antimicrobial agents from the cell. Inhibiting these pumps increases the retention and efficacy of antimicrobial components within the microbial cell (Sharma et al. 2019). Peppermint and cinnamon EOs have shown promise in this regard, enhancing the efficacy of antibiotics against resistant strains (Özakar et al. ).
- Membrane disruption: EOs can disrupt microbial cell membranes, making the cells more permeable to other antimicrobial agents in the mixture (Zengin and Baysal 2014). This disruption can lead to increased leakage of cell contents and eventual cell death. EOs like carvacrol (found in oregano) and cinnamaldehyde (from cinnamon) disrupt the cell membranes of bacteria, leading to leakage of cellular contents and cell death. Studies have shown that these oils can create membrane pores, making bacteria more susceptible to antibiotics (Yap et al. 2014; Akacha et al. 2022a; Zhao et al. 2023; Ellouze et al. 2024). Mechanistically, these compounds rapidly partition into phospholipid bilayers and modulate bilayer physical properties in a concentration‐dependent manner. At low to moderate concentrations, they increase membrane fluidity (decrease lipid order), as shown by reduced fluorescence anisotropy and a lowered and broadened main phase transition in differential scanning calorimetry (DSC); these changes increase lateral diffusion and can create transient defects. Liposome leakage and black lipid membrane experiments indicate that such defects permit nonspecific leakage of small solutes rather than the formation of a single discrete proteinaceous channel. The increased permeability causes rapid dissipation of membrane potential (Δψ) and proton motive force measurable by potentiometric dyes, which inhibit ATP synthesis and secondary transport processes. In parallel, the altered lipid matrix impairs the function of integral membrane proteins (transporters, ATPases), either by changing local bilayer thickness and lateral pressure or by promoting protein misfolding; at higher concentrations, prolonged exposure produces irreversible membrane rupture and cytoplasmic leakage. Ultrastructural evidence from TEM/SEM (Famuyide et al. 2020; Akacha et al. 2024a) shows blebbing, membrane detachment, and cell lysis in treated cells, thereby linking the biophysical measurements to morphological damage. Together, these data support a model in which carvacrol and cinnamaldehyde act primarily through bilayer partitioning, this increased fluidity and defect formation resulting in loss of Δψ and membrane protein dysfunction, ultimately causing irreversible membrane damage and cell death (De Sousa et al. 2012). The molecular mechanisms governing the interaction of EO components (e.g., eugenol, carvacrol, and cinnamaldehyde) with microbial membranes, along with their corresponding experimental signatures and recommended confirmation assays, are summarized in Table 2.

**TABLE 2:** Mechanistic pathways and key experimental signatures demonstrate the interaction between essential oil constituents with microbial targets.

Mechanism	Typical experimental signatures	Representative essays/ evidence	Notes	References
Direct enzyme inhibition (e.g., β‐(1,3)‐glucan synthase)	↓Vmax and/or ↑Km; altered Lineweaver–Burk slopes; measurable KD	Enzyme kinetics; Lineweaver–Burk plots; ITC / MST; docking; site‐directed mutagenesis	Distinguish mixed/non‐competitive vs competitive; check reversibility	(Zhai et al. 2025).
Membrane partitioning/fluidization	↓fluorescence anisotropy; DSC transition broadening; liposome leakage	DPH/TMA‐DPH anisotropy; DSC; liposome leakage assays	Early stage: reversible fluidisation; late stage: irreversible rupture	(Keksel et al. 2019).
Pore/defect formation and permeability loss	Rapid uptake of small dyes; conductance traces showing stochastic defects	Liposome leakage; black lipid membrane; ion conductance assays	Typically non‐proteinaceous, transient pores at low conc.	(Margheritis et al. 2023).
Loss of membrane potential & bioenergetic collapse	Rapid Δψ dissipation; ATP depletion; impaired transport	DiSC3(5), DiOC dyes; ATP assays; transport assays	Correlate with bactericidal vs bacteriostatic outcome	(Suzuki et al. 2003).
Membrane protein dysfunction/denaturation	Reduced membrane enzyme activities; proteomic loss in the membrane fraction	Membrane enzyme assays; proteomics; western blot of membrane proteins	Often secondary to lipid property changes	(Chandramouli and Qian 2009).
Ultrastructural damage	Blebbing, detached membrane, cytoplasmic leakage, lysis	TEM, SEM (representative micrographs)	Use to visually corroborate biophysical and viability data	(Famuyide et al. 2020).

*Abbreviations*: ATP, adenosine triphosphate; DiOC2(3), 3,3'‐diethyloxacarbocyanine iodide; DiSC3(5), 3,3'‐dipropylthiadicarbocyanine iodide; DPH, 1,6‐diphenyl‐1,3,5‐hexatriene; DSC, differential scanning calorimetry; ITC, isothermal titration calorimetry; KD, dissociation constant; Km, Michaelis–Menten constant; MST, microscale thermophoresis; SEM, scanning electron microscopy; TEM, transmission electron microscopy; TMA‐DPH, trimethylammonium–1,6‐diphenyl‐1,3,5‐hexatriene; Vmax, maximum reaction velocity; Δψ, membrane potential.

**FIGURE 4:**
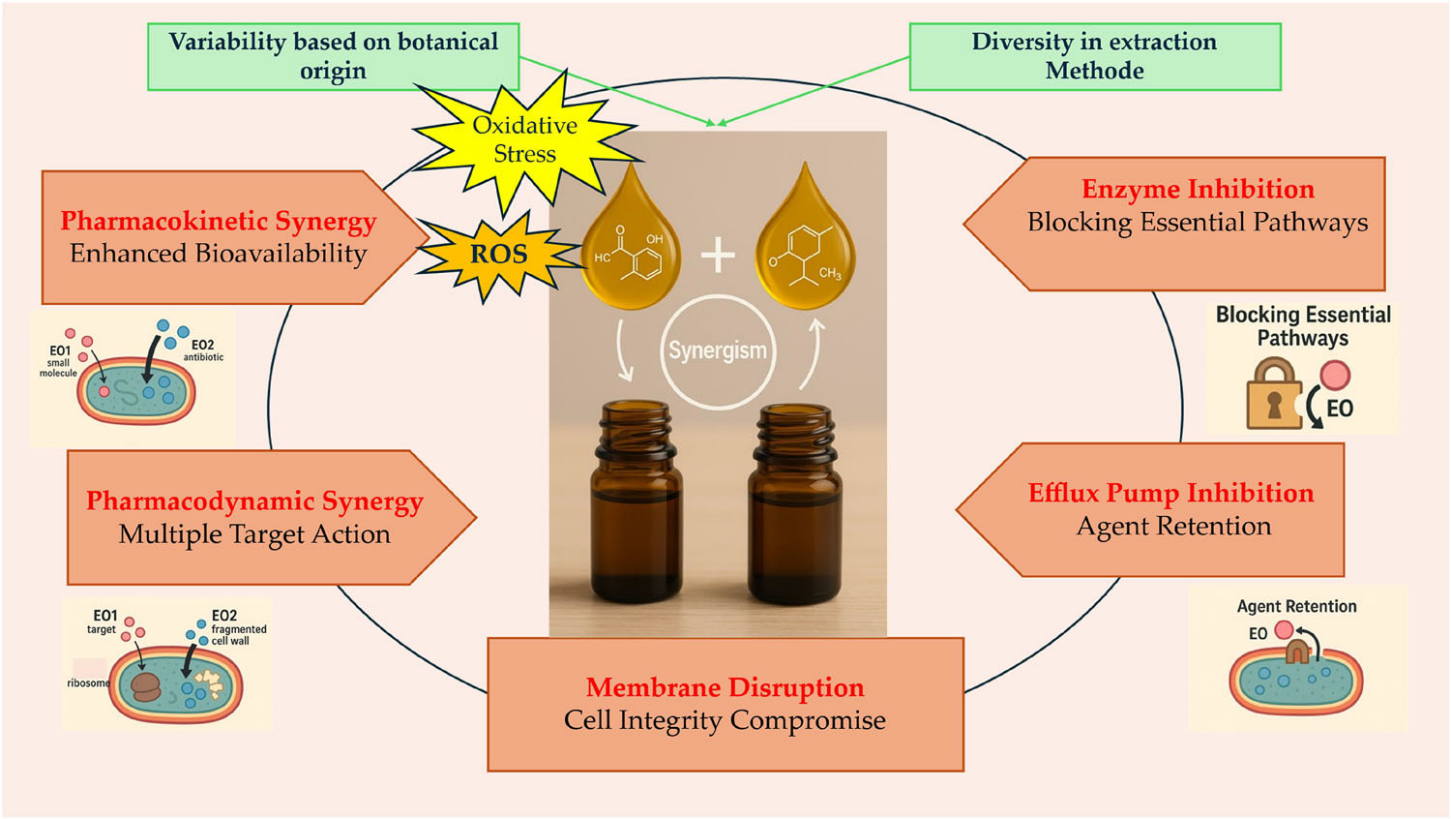
Key mechanisms of essential oil synergy in antimicrobial activity.

Some components of EOs can induce oxidative stress in microbial cells and generate reactive oxygen species (ROS) that damage cellular components (Juan et al. 2021). For instance, eucalyptus and tea tree oil are known for their ability to generate ROS that damage bacterial components. Furthermore, EOs like peppermint have demonstrated significant antioxidant activity that can also inhibit bacterial growth (Bhavaniramya et al. 2019). The overall antimicrobial effect is enhanced with other compounds targeting different cellular processes. Understanding these mechanisms is crucial for rationally developing EO blends that maximize the synergistic effects, leading to a more effective and efficient antimicrobial treatment.  The synergistic interaction between EOs is presented in Figure 4, the schematic illustration below summarizes the main mechanisms underlying this synergistic antimicrobial effect.

## 6 Exploring the Complex Antibacterial Interactions of EOs

Essential oils (EOs) have recently gained attention as possible alternatives to harmful chemical products in many areas (Akacha et al. 2024a). Researchers have investigated the antibacterial effects of EOs against *E.coli* and *S. aureus* (which are considered potential food safety and public health pathogens) (Akacha et al. 2022a; Kačániová et al. 2024). Nevertheless, researchers have yet to explore the full extent of their antibacterial capabilities. Essential oils have a complex composition with numerous chemical constituents that can lead to varying antibacterial activity depending on the species and formulation. While the antibacterial properties of individual essential oils have been extensively studied, there are few reports on the combined effects of multiple essential oils. There is also a lack of research investigating different EO‐based treatments against target bacteria. Essential oils are volatile and lipophilic mixtures of plants that often contain more than a thousand compounds. This composition can vary depending on the species, harvest area, extraction process, and post‐treatment (Dima and Dima 2015). The main classes of compounds found in EOs, including monoterpenes, diterpenes, sesquiterpenes, and aromatic compounds, typically contain one or more cyclic structures within their molecular framework (Siddiqua et al. 2015). These structural features strongly influence their physical and chemical properties, such as volatility and solubility. The distinct characteristics of each EO can therefore result in variations in their water solubility and biological activity. When evaluating the combined antibacterial effects of EOs, it is essential to consider the nature of their interactions, which may involve mutual enhancement (synergism), mutual inhibition (antagonism), or no interaction (independence). Furthermore, accurate calculation and expression of experimental parameters, such as total concentrations and component ratios, are crucial for reliable interpretation of synergistic or antagonistic effects (Yap et al. 2014; Ben Hsouna et al. 2017; Akacha et al. 2025).

Investigating the multifaceted interactions among 20 different essential oil components against *E. coli* and *S. aureus* suggested that components without direct antibacterial activity could act as antagonists (Aelenei et al. 2016). Although this approach provides theoretical predictions of interaction types, further criticism or additional investigations are still required. In contrast to these complex mixtures, simpler and more manageable EO blends that focus on the essential characteristics of individual EOs and their diverse phytochemical origins are considered.

### 6.1 Case Studies and Applications

The following cases verify the above‐mentioned complex interactions through mixture design, providing a basis for the practical application of EO blending. The investigation of EOs for their antibacterial properties highlighted how mixture design methods, such as the extended simplex centroid design and the extended simplex lattice design, can systematically optimize EO combinations for natural preservation. Ouedrhiri et al. (2016) investigated the combined effect of *Origanum compactum*, *Origanum majorana*, and *Thymus serpyllum*. The antibacterial response was modelled using disk diffusion and microdilution methods by a cubic polynomial equation detailing the oils' individual and interactive effects. The results showed significant synergistic effects, especially against gram‐positive bacteria such as *Bacillus subtilis* and *S. aureus*, suggesting potential applications of these EO blends in natural food preservation.

In a related study, Ouedrhiri et al. (2022), investigated the antibacterial potential of EOs extracted from *Myrtus communis* (*M. communis*), *Artemisia herba‐alba* (*A. herba‐alba*), and *Thymus serpyllum* (*T. serpyllum*) using an extended simplex centroid mixture design. The authors aimed to identify the most effective combinations of these oils against *B. subtilis*, *S. aureus*, and *E. coli*. Gas chromatography–mass spectrometry analysis showed that *M. communis* oil was mainly composed of myrtenyl acetate, linalool, and 1,8‐cineole; A. herba‐alba oil was rich in piperitone; and *T. serpyllum* oil contained high amounts of *p*‐cymene, *γ*‐terpinene, and thymol. Using a cubic polynomial model and least‐squares regression, the study described the interactions between individual and combined oils, with statistical validation by ANOVA confirming the significance and predictive reliability of the model. The results showed that *T. serpyllum* exhibited the strongest individual antibacterial activity, particularly against *S. aureus* and *E. coli*, while the ternary mixture of *M. communis*, *A. herba‐alba*, and *T. serpyllum* produced the most potent inhibitory effect against *B. subtilis*. The analysis revealed significant synergistic effects between *M. communis* and *T. serpyllum*, as well as between *M. communis* and *A. herba‐alba*, whereas the combination of *A. herba‐alba* and *T. serpyllum* displayed partial antagonism towards *E. coli*. These findings highlight that the antibacterial activity of EOs is not merely additive but depends strongly on the interactions among their constituents. The study further emphasized that optimal efficacy can be achieved by fine‐tuning the proportions of each oil rather than relying on individual components, thereby demonstrating the potential of mixture design as a powerful tool for developing synergistic and broad‐spectrum natural antibacterial formulations.

Another study performed by Zieniuk and Bętkowska (2021) investigated the antimicrobial properties of the EOs of tea tree (*Melaleuca alternifolia*), rosewood (*Aniba rosaeodora*), and lavender (*Lavandula hybrida*). Using an extended simplex lattice design, they tested each oil individually against *E. coli*, *L. monocytogenes*, and *Rhodotorula mucilaginosa*. Their results showed that tea tree oil, known for its high phenolic content, had the most potent antibacterial activity. Optimization of the blends also showed that combinations with higher levels of tea tree oil were particularly effective against the tested microorganisms, making it an essential candidate for natural food preservation due to its high efficacy.

The EO combinations study also used the oils of *Citrus sinensis*, *Pistacia lentiscus*, and *Eucalyptus citriodora* to inhibit the growth of *Candida albicans*, *Staphylococcus aureus*, *E. coli*, *Salmonella enterica*, and *B. cereus*, using a simplex‐centric mixture design. This design showed the additive, synergistic, and antagonistic interactions in triangular diagrams and enabled the researchers to find the ideal EO ratio. Of note, compounds such as *d*‐limonene in sweet orange and citronellal in lemon eucalyptus enhanced antibacterial efficacy, significantly reducing MICs in combined formulations compared to single oils. This result underlines the potential of these EOs mixtures to serve as effective natural preservatives (Al‐Mijalli et al. 2023).

Another study of Akacha et al. (2024b) investigated a mixture of *α*‐pinene, *α*‐terpineol, and 1,8‐cineole using an elevated simplex centroid mixture design. Chemometric analysis and RSM were used to fine‐tune the antibacterial properties of the mixture, with optimal ratios achieving MICs between 0.31 and 1.85 mg/mL. A mixture composed of equal parts (0.33 each) exhibited synergistic antibacterial activity against *B. cereus*, reinforcing the potential of essential oil combinations for food preservation. Similarly, Ellouze et al. (2024) demonstrated that combinations of *Citrus aurantium* EOs, particularly those rich in linalool and linalyl acetate, showed enhanced antibacterial effects, with binary and ternary mixtures reducing the MIC against *E. coli* to as low as 0.21 mg/mL. Fractional inhibitory concentration index (FICI) analysis confirmed the synergistic interactions, indicating the potential of the blends to inhibit spoilage bacteria in raw meat and demonstrating the value of blend designs in optimizing natural preservatives to replace synthetic agents in food safety.

Overall, mixture‐design approaches (extended simplex centroid and simplex lattice designs), combined with GC–MS chemical profiling and cubic‐polynomial/RSM modelling validated by ANOVA, provide a robust framework for optimising essential oil formulations with antibacterial properties. Chemical characterisation links observed bioactivity to major constituents (e.g., thymol, *p*‐cymene, 1,8‐cineole, linalool, piperitone, and *d*‐limonene), and the results clearly indicate that efficacy is not merely additive: specific binary and ternary proportions can produce pronounced synergistic effects that depend on both composition and target organism (Al‐Mijalli et al. 2023). Optimized blends in these studies substantially reduced MICs/CIMs against key foodborne and spoilage microbes (*B. subtilis*, *S. aureus*, *E. coli*, and *Candida* spp.), highlighting strong potential for natural food preservation. To translate these promising findings into practical applications, however, further studies should evaluate performance in real food matrices, sensory and stability impacts, toxicological safety, and suitable delivery systems. Altogether, mixture‐design methods emerge as powerful, data‐driven tools for developing tailored, broad‐spectrum essential oil formulations for safer, more natural food preservation.

### 6.2 Current Patents and the Future Role of Mixture Design in EO Antibacterial Research

There are some patents describing the use of mixtures of EOs that work together to enhance the antibacterial effect. One patent uses a blend of EOs such as thymol, eugenol, menthol, and carvacrol in dental care products to reduce bacterial growth, demonstrating the versatility of essential oil blends for oral health. In addition, the synergistic essential oil compositions in which EOs are blended with antibacterial agents such as zinc salts and plant antioxidants to enhance personal care products' antibacterial and anti‐inflammatory properties were also patented by Prencipe et al. (2018). This patent demonstrates the diverse potential of EO combinations in developing effective antibacterial formulations in various industries.

However, none of the current patents use a statistical blend design as a powerful optimization tool as part of the approach to develop and refine EO combinations for maximum antibacterial efficacy. As it allows the precise identification of the optimal ratio of components, blend design has excellent potential for future exploration where statistical approaches could improve the predictability and efficacy of essential oil blends in antibacterial applications. This could provide a structured route to develop effective natural antimicrobials and represent an innovative step forward in food preservation and therapeutic formulations.

## 7 The Antioxidant Activity of Essential Oil Blends and Approaches to Experimental Design

Essential oil constituents, particularly phenolic monoterpenes (e.g., thymol, eugenol, and carvacrol) and certain terpenoids (e.g., citral and α‐pinene), exert antioxidant effects through multiple biochemical pathways. At the molecular level, phenolics neutralize free radicals via hydrogen atom transfer (HAT) or single‐electron transfer (SET), yielding stabilized phenoxyl intermediates (Costa et al. 2019; Özakar et al). For example, thymol and eugenol can donate hydrogen atoms from their aromatic ^–^OH groups to quench peroxyl (ROO•) and other reactive oxygen species (ROS), thereby interrupting lipid peroxidation chain reactions. Many essential oil phenols also chelate transition metal ions (e.g., Fe^2^⁺, and Cu^2^⁺), forming inert complexes that block Fenton reactions and hydroxyl radical (^•^OH) generation (Costa et al. 2019). These radical‐scavenging and chelating actions stabilize cellular membranes by preventing peroxidative damage to phospholipids. Concurrently, EO components modulate redox‐sensitive signalling: phenolic essential oils such as eugenol inhibit Kelch‐like ECH‐associated protein 1 (Keap1), freeing nuclear factor erythroid 2‐related factor 2 (Nrf2) to accumulate and translocate to the nucleus. Activated Nrf2 binds to antioxidant response elements (ARE) in DNA, promoting the transcription of phase II antioxidant genes such as superoxide dismutase (SOD), catalase (CAT), glutathione peroxidase (GPx), heme oxygenase‐1 (HO‐1), and NAD(P)H: quinone oxidoreductase‐1. The upregulated enzymes enhance ROS detoxification; for example, SOD converts O_2_^•–^ to H_2_O_2_, which CAT and GPx then reduce to H_2_O, thereby reinforcing cellular redox homeostasis (Wang et al. 2021). Recent studies confirm that nonphenolic monoterpenes such as *α‐*pinene and citral also activate Nrf2‐dependent gene expression in cells.

**FIGURE 5:**
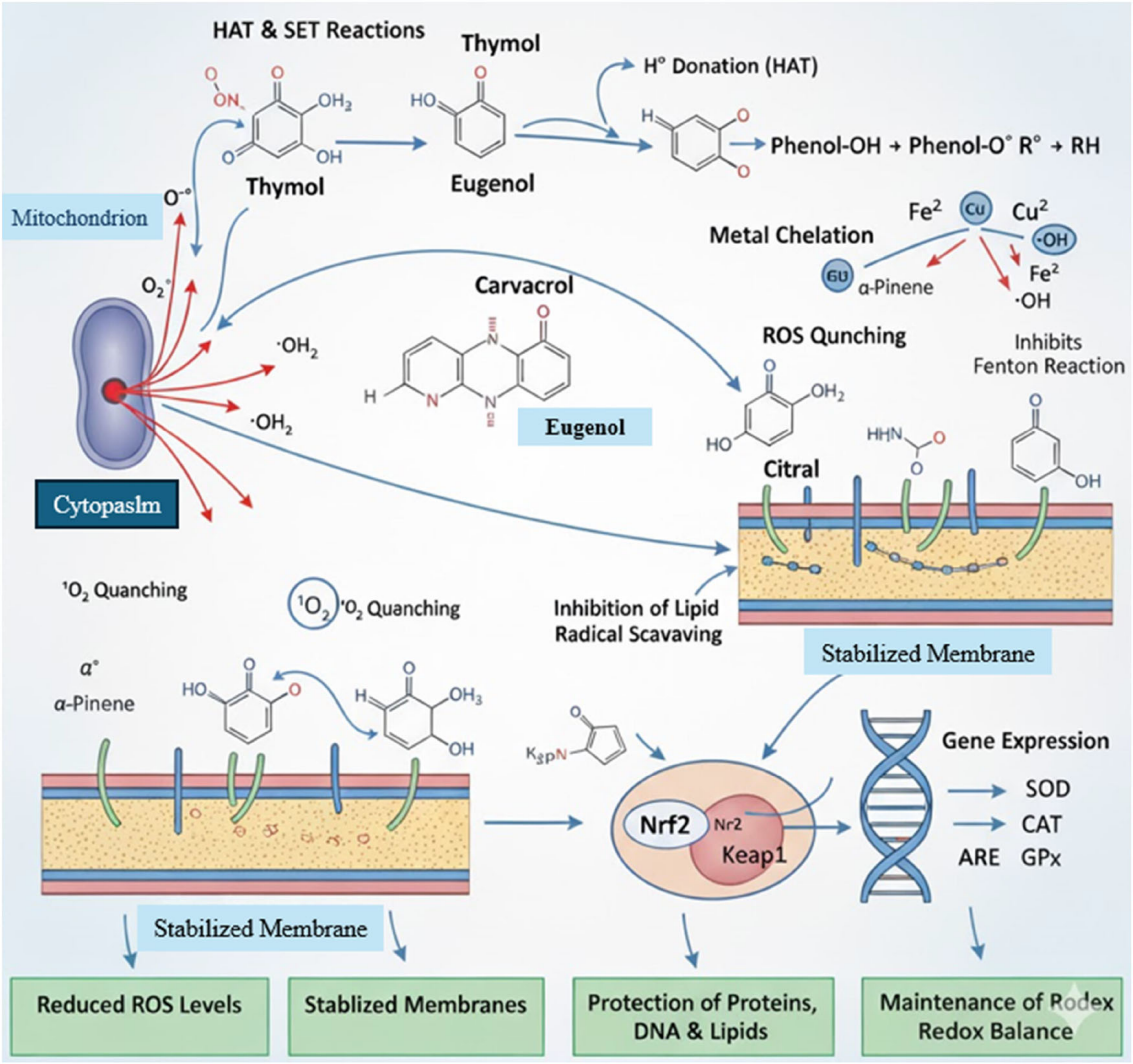
Antioxidant mechanisms of essential oils in cellular protection. HAT: hydrogen atom transfer; SET: single electron transfer; ROS: reactive oxygen species; O_2_^−^•: superoxide anion radical; •OH: hydroxyl radical; H_2_O_2_: hydrogen peroxide; ^1^O_2_: singlet oxygen; Fe^2^⁺: ferrous iron; Cu^2^⁺: cupric copper; Nrf2: nuclear factor erythroid 2‐related factor 2; Keap1: Kelch‐like ECH‐associated protein 1; ARE: antioxidant response element; SOD: superoxide dismutase; CAT: catalase; and GPx: glutathione peroxidase.

The schematic in Figure 5 begins with mitochondrial ROS generation (electron transport leakage produces O_2_^•–^, which is partially converted to H_2_O_2_ by endogenous SOD). These ROS diffuse into the cytosol and threaten membrane integrity. Essential oil molecules are depicted intercepting ROS at several points: they directly neutralize radicals (red arrows from O_2_^•–^ and ^•^OH to EO icons) and bind Fe^2+^ (arrow to an EO–Fe complex) to prevent ^•^OH formation. At the lipid bilayer, EO components are shown preventing the propagation of lipid peroxidation (the membrane remains intact). Simultaneously, EOs disrupt the Keap1–Nrf2 complex in the cytosol, allowing Nrf2 to enter the nucleus. Inside the nucleus, Nrf2 binds ARE sequences and induces antioxidant genes (SOD, CAT, GPx, HO‐1, etc. are labelled). The translated enzymes (depicted in the cytosol) further detoxify remaining ROS and repair oxidative lesions. Thus, the Figure 5 flows from mitochondrial ROS, through direct chemical quenching and membrane protection, to activation of the Keap1/Nrf_2_ pathway and antioxidant gene expression illustrates the coordinated mechanism by which essential oils mitigate oxidative stress (Zhao et al. 2019; Zeng et al. 2023).

The standard practice is extended simplex centroid and simplex lattice designs for 3–4 oils. In a simplex centroid, pure oils, binary equivalent mixtures of all compositions, and ternary (quaternary) centroids are tested, usually with replicates of the center point to determine errors (Zieniuk and Bętkowska 2021). For example, Soukaina et al. (2025) used an extended simplex centroid for three EOs (*Thymus serpyllum, Mentha pulegium*, and *Mentha piperita*) with 10 runs with replicates. The mixture proportions (which add up to 1) are varied, and the reactions are modelled by polynomial equations. Model fit is checked by ANOVA, *R^2^*, goodness‐of‐fit tests, and so on, and the models usually have an R^2^ of >90%. Optimization then makes use of desirability functions or contour plots to find the blend with the highest antioxidant effect. Some studies also employed D‐optimal designs or multifactor RSM, where components or ratios are constrained (Akacha et al. 2024b; Soukaina et al. 2025).

### 7.1 Optimization of EO Blends for Enhanced Antioxidant Activity

Essential oils cover a wide range of plant sources, but the Lamiaceae species thyme, oregano, basil, rosemary, sage, marjoram, and mint are the best known, along with *Cymbopogon* spp. (lemongrass, citronella), *Carum carvi, Acorus calamus* and others (Ben Hsouna et al. 2022b; Akacha et al. 2024a). The antioxidant effect is primarily the result of phenolic monoterpenes such as thymol, carvacrol, eugenol, and linalool, as well as phenylpropenes such as eugenol and safrole (Costa et al. 2019; Akacha et al. 2023b). Therefore, *Thymus* and *Origanum* oils, which are rich in thymol and carvacrol, are relentlessly sought after for their intense activity, while *Cinnamomum*, *Satureja*, *Rosmarinus*, and *Ocimum* species are relentlessly questioned for their potent antioxidant activity (Bhavaniramya et al. 2019; Taieb Bouteraa et al. 2024; Akacha et al. 2025). The assays used are primarily radical scavenging assays, including (1) 1,1‐diphenyl‐2‐picrylhydrazyl (DPPH), wherein the majority of studies employ IC_50_ or % inhibition; (2) 2,2'‐azinobis‐(3‐ethylbenzothiazoline‐6‐sulfonic acid (ABTS•+) scavenging, often complemented with DPPH; (3) ferric reducing antioxidant power (FRAP), often utilized in multiresponse formats; and (4) total antioxidant capacity. Most of the studies validate their blends against reference standards such as BHT or ascorbic acid (Basavegowda and Baek 2021).

For instance, Torres Neto et al. (2024) demonstrated that a lemongrass and oregano blend was as good as butylated hydroxytoluene (BHT) in preventing trout fillet oxidation. Some authors report absolute scavenging percentages, such as Itam et al. (2021), with 88.1% DPPH inhibition, whereas others present IC_50_ values in µg/mL, where the lower the value, the higher the activity.

The basic observation throughout the literature is that optimized EO mixtures always work better than single oils, often via synergistic mechanisms where the measured activity is higher than the weighted average of the individual components. For example, using DPPH and ABTS assays, Benkhaira (2021), reported that a high‐quality blend of lemongrass (20%), caraway (53%) and calamus (27%) had an IC_50_ determined via DPPH of 183.9 µg/mL, which was better than pure BHT (191.1 µg/mL), while a compromise mixture (18/48/34%) simultaneously optimized both DPPH and ABTS activities (IC_50_ values of 187.8 and 64.6 µg/mL, respectively), suggesting a synergistic effect most likely due to complementary chemistry.

Similarly, Soukaina et al. (2025) experimented with a mixture design involving *Thymus serpyllum, Mentha pulegium*, and *Mentha piperita* and showed that thyme was the most active individual but that the ternary mixture (39% thyme, 28% pennyroyal, 33% peppermint) acquired an experimental DPPH‐IC_50_ of 0.98 µg/mL, lower than the calculated 1.25 µg/mL, thus establishing synergistic potency. Although binary thyme + pennyroyal was extremely synergistic and the ternary model indicated a weak antagonistic effect, the optimal mix improved antioxidant activity and successfully protected olive and argan oils from oxidation. In food applications, Torres Neto et al. (2024), contrasted a combination of three oils with trout fillets and found that the best combination (66% lemongrass, 34% oregano, with thyme reduced to 0%) exhibited maximum DPPH and FRAP activity and, at 2000 ppm,, was as efficient as BHT in inhibiting lipid and protein oxidation, exhibiting additive and synergistic antioxidant activity at intermediate concentrations. Gheisary et al. (2025) optimized blends of *Cuminum cyminum, Origanum vulgare*, and *Salvia officinalis* with desirability functions for DPPH and FRAP, the best blend (∼15% cumin, 74% oregano, 11% sage) showing 88.1% inhibition of DPPH and FRAP ≈ 11.36 µmol Fe(II)/g, much greater than any of the individual oils, with carvacrol of oregano having the most prevalent impact. In a similar vein, Elbouzidi et al. (2024) optimized blends of *Lavandula dentata*, *Rosmarinus officinalis*, and *Myrtus communis* and determined that the best DPPH blend (20/50/30%) had an IC_50_ of ≈71.2 µg/mL, and the best ABTS ratio (∼18/50/32%) had an IC_50_ of ≈44.4 µg/mL, both significantly greater than individual oils, which they attributed to synergistic phenolic content, proposing these blends for food and pharmaceutical applications.

Other studies confirm these findings and show that thymol‐ and carvacrol‐rich oils such as thyme, oregano, and marjoram often dominate in optimal blends, although combining them with others such as mint, lemongrass, or parsley broadens the antioxidant spectrum (Table 3). An extensive survey of 423 EOs confirmed that Lamiaceae and Myrtaceae have the highest DPPH activity, which is consistent with their abundance in mixtures (Assaggaf et al. 2024).

**TABLE 3:** Summary of mixture‐design studies on EO blends for antioxidant activity.

Study (EOs)	Mixture design	Assays	Optimal blend	Outcome	Synergy	Ref.
*C. flexuosus*, *C. carvi*, *A. calamus*	Augmented simplex‐centroid (3 EOs; 12 runs)	DPPH, ABTS	20% lemongrass (*C. flexuosus*), 53% caraway (*C. carvi*), 27% calamus (*A. calamus*)	DPPH‐IC_50_ ≈183.9 µg/mL (vs BHT 191.1)—better than any single oil; simultaneous DPPH/ABTS optimum at ∼18/48/34%.	Synergy of components	(Assaggaf et al. 2024).
*T. serpyllum*, *M. pulegium*, *M. piperita*	Augmented simplex‐centroid (3 EOs; 12 runs)	DPPH (IC50)	39% thyme, 28% pennyroyal, 33% peppermint (*Thymus/Mentha*)	Experimental DPPH‐IC_50_ ∼0.98 µg/mL vs model 1.25 µg/mL.	Thyme and pennyroyal binary showed strong synergy; the ternary interaction term was antagonistic, highlighting complex interplay	(Ouedrhiri et al. 2016).
*O. vulgare*, *T. vulgaris*, *C. citratus*	Augmented simplex‐centroid (3 EOs; 12 runs)	DPPH, FRAP	66% lemongrass (*C. citratus*), 34% oregano (*O. vulgare*), 0% thyme (*T. vulgaris*)	Maximal DPPH and FRAP, matching the effect of 100 ppm BHT on fish fillets.	Inhibition of lipid and protein oxidation during storage, demonstrating effective synergy at lower doses	(Gheisary et al. 2025).
*O. basilicum*, *C. nardus*, *J. virginiana*, *T. vulgaris*	Simplex‐lattice (4 EOs; 15 runs)	DPPH (EC50)	54.4% citronella (*C. nardus*), 33.0% thyme, 9.2% cedarwood (*J. virginiana*), 3.4% basil	The best blend was predicted and matched experimentally, achieving EC_50_ = 0.65 mg/mL.	“Interesting synergy”	(Oliveira et al. 2024).
*Cuminum cyminum*, *O. vulgare*, *S. officinalis*	Simplex‐lattice (3 EOs; 10 runs)	DPPH, FRAP	≈15% cumin (*C. cyminum*), 74% oregano, 11% sage (*S. officinalis*)	DPPH 88.1% inhibition, FRAP ≈11.36 µmol Fe (II)/g at optimum, far above single oils. Oregano's carvacrol drove the high antioxidant effect.	Multiresponse desirability yielded this as the best formulation	(Gheisary et al. 2025).
*L. dentata*, *R. officinalis*, *M. communis*	Augmented simplex‐centroid (3 EOs; 12 runs)	DPPH, ABTS	20% lavender, 50% rosemary, 30% myrtle (for DPPH); ∼18/50/32% for ABTS	Achieved DPPH‐IC50 ∼71.2 µg/mL and ABTS‐IC50 ∼44.4 µg/mL, significantly better than any pure EO.	Optimal ratios reflect complementary phenolics	(Elbouzidi et al. 2024).

*Note*: Selected examples from 2018–2025; optimal blends given as % *v/v*; major assays listed.

In terms of general trends, almost all optimized mixtures show improved degradation of DPPH radicals, which ABTS and FRAP confirm. Increasingly, optimization of multiple reactions is being used, such as in Najafi et al. (2022), who maximized DPPH and ABTS simultaneously. While most reports emphasized synergy (the activity of the mixture is greater than the expected additive effect), some studies, such as Soukaina et al. (2025), have found partial antagonism (negative ternary coefficients), highlighting the complexity of EO interactions where one oil can dominate or inhibit another. To address this issue, most authors apply desirability functions to identify optimal trade‐offs, often integrating multiple tests. Overall, the optimization confirms literature reports that combining hydrogen‐donating phenols such as thymol with electron‐rich terpenes enhances the radical scavenger through complementary mechanisms, providing strong evidence that EO blends can be strategically formulated to achieve superior antioxidant activity.

### 7.2 Limitations and Points for Improvement

While the blend design studies reviewed here provide compelling evidence for the enhanced antioxidant activity of EO blends, there are several limitations that deserve careful attention. First, chemical variability between individual essential oils (due to differences in plant source, harvest time, extraction method, storage, and chemotype) often results in non‐reproducibility between studies, making it difficult to generalize optimal blends beyond the specific samples used. Secondly, most studies rely solely on in vitro radical scavenging assays, which, while applicable for screening, do not necessarily reflect antioxidant behaviour in more complex matrices or under oxidative stress conditions. Third, although mixture designs help optimize blends, they may not fully capture real‐world constraints (e.g., volatile compound interactions, stability over time, sensory attributes, toxicity, regulatory limits, and cost). Fourth, synergy is often inferred simply by comparing the observed activity vs. the expected additive effect. However, mechanisms of action (molecular level) are seldom probed; thus, the chemical basis of synergy (or antagonism) remains underexplored. Complementary experimental approaches reported in the literature include: (i) enzyme kinetics and biochemical inhibition assays to quantify direct effects on catalytic activity; (ii) binding assays to detect ligand enzyme interactions and estimate $K_{D}$; (iii) biophysical membrane assays to measure changes in lipid order and permeability; (iv) membrane potential assays to monitor Δψ loss; and (v) ultrastructural imaging to visualize morphological damage

Where only one class of evidence is available, we note this limitation and refrain from asserting a single exclusive molecular pathway. Finally, the lack of long‐term stability studies (oxidative stability, shelf life, component degradation) and scaled‐up validation limits the translation of blend formulations into practical products.

## 8 Future‐Oriented Research Directions for EOs Optimization and Real‐World Efficacy

Future directions of research on EO optimization and efficiency in the real world: Future studies should investigate a broader range of microbial species, including other foodborne pathogens, spoilage microorganisms, and antibiotic‐resistant pathogens, to further elucidate the efficiency of EO mixtures in most real‐world applications in food safety, medicine, and agriculture. The elucidation of the molecular mechanisms of antibacterial, antioxidant, and synergistic activities of EO mixtures is of great importance. More advanced imaging, genomic, and proteomic techniques could be used in research to find out how EO interacts with microbial cell membranes, enzymes, efflux pumps, and ROS to enhance antimicrobial and free radical scavenging activity further. Research should also evaluate how antioxidant compounds such as thymol, carvacrol, and eugenol regulate oxidative stress pathways, inhibit lipid peroxidation, and protect biomolecules from oxidative degradation, as these factors are critical for food shelf life and therapeutic efficacy. Stability studies would investigate the antioxidant ability of EO under different storage conditions (e.g., different temperatures, humidity, light, and pH) to determine optimal conditions for maintaining radical scavenging ability and efficacy in various application scenarios.

Subsequent studies could explore encapsulation methods such as nanoemulsions, cyclodextrin inclusion complexes, or liposomes that not only protect the EO from degradative environmental factors but also preserve the antioxidant effect and support‐controlled release. These strategies can prolong the antimicrobial and antioxidant effect of food packaging, dietary supplements, or cosmetics and reduce the amount of EOs required for an effective impact. Beyond binary or ternary blends, future research can extend blend design strategies to multi‐component EO formulations and investigate how different combinations of three or more EOs contribute to synergistic, additive, or antagonistic effects for both antibacterial and antioxidant activity and reduce the risk of developing microbial resistance.

Machine learning algorithms can be integrated into mixture design methods to enable more accurate prediction of the best EO mixtures based on algorithms that sift through experimental data from DPPH, ABTS, and FRAP tests to achieve effective selection of mixtures with maximum radical scavenging capacity and antibacterial activity with minimal large‐scale experimental trials. At the same time, the experiments must also include sensory and toxicity analyses to ensure that the EO formulations with high antioxidant activity are palatable and safe for consumption, especially in food and cosmetics, and to avoid potential irritation or odour nuisance associated with EO preparations.

To enhance the translational impact of EOs research, future studies should prioritize approaches that move beyond incremental replications of binary or ternary blends towards integrative, application‐driven strategies. Multi‐component blending enables exploration of high‐dimensional synergy spaces that traditional pairwise studies cannot access; its innovation lies in combining high‐throughput screening and chemometric pre‐filtering with optimal experimental designs (such as D‐optimal or augmented simplex methods) to identify non‐obvious, higher‐order interactions. Encapsulation and delivery systems should progress from ad hoc demonstrations to rational carrier engineering, such as co‐encapsulation, stimuli‐responsive release, and matrix‐specific release profiling, to protect labile constituents and deliver active doses where required. Machine learning‐guided optimization should serve as an explainable, data‐efficient layer that prioritizes candidate mixtures for focused design of experiments, using feature‐importance methods to reveal molecular determinants of synergy rather than relying solely on black‐box prediction. The most promising path forward is an integrated pipeline that reduces experimental burden, identifies mechanistic drivers of synergy, and produces formulations ready for real‐world validation.

Furthermore, the efficacy of EO mixtures in terms of antimicrobial activity and inhibition of oxidative spoilage in real‐life applications such as food preservation, crop protection from agricultural insects, and medicine must be verified in the future. For example, their ability to delay lipid and protein oxidation in meat, milk, and edible vegetable oils should be thoroughly tested under industrial conditions. These recommendations provide a roadmap for further development of EO research. They may lead to more stable, effective, and universal natural antimicrobials and antioxidants that can promote sustainability and reduce the use of synthetic chemicals.

## 9 Conclusions

Applying mixture design in essential oil research provides a powerful tool to systematically identify and optimize. Statistical mixture design approaches offer a systematic and effective method for optimizing multi‐component essential oil blends to enhance antimicrobial and antioxidant efficacy. The reviewed studies consistently show that blends identified using these methods often achieve substantially greater bioactivity than individual oils, reflecting synergistic interactions among components. Chemical profiling combined with statistical modelling in these studies confirms that the enhanced efficacy is linked to specific interactions among major constituents, resulting in pronounced synergistic effects depending on blend composition and target organism.

Across diverse case studies and microbial targets, optimized essential oil blends displayed broad‐spectrum antimicrobial activity and enhanced antioxidant performance. These outcomes validate mixture design as an effective framework for developing natural preservative formulations. In all cases, blend formulations outperformed their single oil counterparts, underscoring the practical value of this approach. Together, these outcomes affirm that statistical blend optimization is a robust strategy to harness the inherent synergistic potential of essential oils for producing safer, eco‐friendly antimicrobial and antioxidant agents. In summary, the accumulated evidence confirms that mixture design can reliably guide the formulation of high‐efficacy natural products to improve food safety, shelf life, and health applications.

## Author Contributions

**Boutheina Ben Akacha**: conceptualization, writing – original draft, writing – review and editing. **Miroslava Kačániová**: writing – review, and editing. **Wirginia Kukula‐Koch**: writing – review and editing. **Sandra Cabo Verde**: writing – review and editing. **Joana Madureira**: writing – review, and editing. **Wojciech Koch**: writing – review, and editing. **Rania Ben Saad**: writing – review, and editing. **Monika Michalak**: conceptualization, visualization, writing – review, and editing. **Stefania Garzoli**: supervision, writing – review, and editing. **Anis Ben Hsouna**: conceptualization, visualization, supervision, project administration, writing – review, and editing.

## Conflicts of Interest

The authors declare no conflicts of interest.
